# Transfusion of Blood, Report of a Vivisection

**Published:** 1869-12

**Authors:** Prof. Freer

**Affiliations:** Rush Medical College


Article II.—*Transfusion of Blood. Report of a Vivisection, Illustrating Lectures on the Blood*, by Prof. Freer, Rush Medical College. Reported by F. L. Wadsworth, M.D., Chicago, Illinois.

The following account of a vivisection, performed before the class, at Rush Medical College, on the 10th inst., by Dr. Freer, Professor of Physiology and Microscopic Anatomy, may be of value to your readers.

A dog, the weight of which was fourteen pounds avoirdupois, was anæsthetized, the left carotid artery and jugular veins were exposed, a canula inserted into each, and secured by ligatures. Sufficient time was then allowed for the animal to recover his consciousness from the effects of the anæsthetic, when the stop-cock of the canula inserted into the artery was turned, and the blood allowed to flow until sixteen ounces were extracted and the force of the heart's pulsations so reduced that no more could be drawn from the artery with the animal lying horizontally upon his right side. Respiration now gave evidence of syncope, the limbs were rigid, the jaws set, death was impendent.

Meantime, a portion of the blood drawn had been defibrinated and kept at its normal temperature by a warm water bath. It was now (twenty minutes from the time it commenced to flow from the artery) passed through the canula, by means of a syringe, into the jugular vein. In this manner seven ounces of defibrinated blood were returned to the system of the animal.

On the introduction of the first ounce there was observed decided evidence of increased vigor. During the injection of the second ounce, by some defect in the apparatus, air was admitted into the vein. Instantly the animal succumbed; a tremor affected his entire system, and respiration ceased. Prof. Freer remarked that the animal's life had been sacrificed by the accident; but by convulsive efforts he regained respiration, and in two or three minutes the effects of the occurrence had disappeared. When the seventh ounce had been introduced there remained little, if any, disturbance of respiration, the heart's vigor was nearly restored, the muscular spasm had subsided, and there was apparently no more disturbance of the system than usually occurs at this period after the administration of chloroform for any ordinary operation.

In eight minutes after the blood had been returned the animal raised his head and observed intelligently; and twelve minutes thereafter he voluntarily raised himself and walked across the room, some twelve or fifteen feet. The day following he was sprightly and playful, and the second day after the operation he traveled nearly a quarter of a mile without any evidence of fatigue whatever.

This *vivisection* occurred during the delivery of a lecture on the blood, by Prof. Freer, and was introduced to illustrate the function of the red blood corpuscles in their immediate relations to respiration and the maintenance of vitality, and to demonstrate the fact that fibrin takes no immediate part in restoration from exhaustive hæmorrhage, serum serving as the vehicle of the vitalizing corpuscle, and that transfusion with defibrinated blood may be performed somewhat leisurely and with impunity, care being taken that the instruments used are nicely adapted for the exclusion of air from the vessels.

